# Evaluation of Strategies for the Development of Efficient Code for Raspberry Pi Devices

**DOI:** 10.3390/s18114066

**Published:** 2018-11-21

**Authors:** Javier Corral-García, José-Luis González-Sánchez, Miguel-Ángel Pérez-Toledano

**Affiliations:** 1 CénitS–COMPUTAEX, Extremadura Supercomputing, Technological Innovation and Research Center, 10071 Cáceres, Spain; joseluis.gonzalez@cenits.es; 2 Computer Science Department, University of Extremadura, 10003 Cáceres, Spain; toledano@unex.es

**Keywords:** Raspberry Pi, efficient code, code optimization, performance optimization

## Abstract

The Internet of Things (IoT) is faced with challenges that require green solutions and energy-efficient paradigms. Architectures (such as ARM) have evolved significantly in recent years, with improvements to processor efficiency, essential for always-on devices, as a focal point. However, as far as software is concerned, few approaches analyse the advantages of writing efficient code when programming IoT devices. Therefore, this proposal aims to improve source code optimization to achieve better execution times. In addition, the importance of various techniques for writing efficient code for Raspberry Pi devices is analysed, with the objective of increasing execution speed. A complete set of tests have been developed exclusively for analysing and measuring the improvements achieved when applying each of these techniques. This will raise awareness of the significant impact the recommended techniques can have.

## 1. Introduction

The emergence of the Internet of Things (IoT) aims to improve quality of life by connecting smart devices, applications, and technologies, empowered by the high volume of smart sensors, radio-frequency identifications, communications and Internet protocols available. In this way, IoT allows all kinds of physical objects to connect to the Internet and interact without the need for human intervention, turning these objects into intelligent devices [1]. However, despite its growth potential it still faces many challenges, mainly related to addressing, routing, standardization, restricted physical capabilities (e.g., energy, processing, and memory), security, privacy, and openness [2].

With regard to IoT sensing, data are collected from objects and sent to a data warehouse, database, cloud or similar, for analysis in order to take specific actions. Thus, single-board computers (SBCs), integrated with sensors, built-in TCP/IP, and security functionalities are currently being extensively used in the development of IoT products. Although they cannot compete with the computing performance of higher-value systems due to limited computational power, they often represent the best option to build inexpensive, mobile and low-power systems because of their compact design, very low cost/performance ratio, and energy consumption. In this way, they are gaining increasing interest in research computing, and the number of ARM processors in these kinds of devices has grown exponentially due to their low power usage.

One of the most dominant SBCs, along with the Arduino, is the Raspberry Pi (RPi) [3]: a low-cost, credit card sized, computer. The RPi was initially created to promote the teaching of basic computer science in schools and developing countries, but has since become one of the most commonly used devices (in 2017, the Raspberry Pi Foundation [3] announced 14 million of their devices had been sold since 2012). It is particularly remarkable for the computing power it offers per watt, allowing essential computing tasks to be performed in a more energy efficient way, consuming less power and saving a notable amount of energy. Mobility also plays an important role in the success of the RPi.

Given its suitability for applications in many areas, the RPi has been used in a large number of research projects, from sensor networks [4,5,6], vehicle active safety systems [7], e-health care [8], big data analytics [9], and image analysis [10], to various other areas. It has also been widely used in educational projects [11], and in the construction of affordable and energy-efficient clusters consisting of up to 300 nodes [12], which partially offsets its lack of computing power.

Thus, this proposal aims to analyse the application of diverse techniques for the efficient writing of programs in Raspberry Pi devices, preserving the semantics of the program to reduce execution times without affecting the original algorithms (and, consequently, the results), with the objective of speeding up the executions. The approach evaluates the execution times obtained through the manual application of these techniques, compared to the automatic optimizations offered by the GNU Compiler Collection (GCC) [13]. The experimental results demonstrate that the proposed techniques achieve significant improvements in runtime, even improving automatic optimizations (offered by the compiler if the techniques are not applied). Thus, it is shown that optimization options provided by GCC do not achieve the same improvements that programmers can achieve when applying these techniques manually. In addition, this proposal provides a set of tests developed to allow the measurement and analysis of these techniques.

The proposal, therefore, is intended, firstly to help programmers to directly write efficient code, and, secondly, to show that the help offered by the compiler is, in some cases, insufficient when it comes to obtaining the best efficiency.

The rest of the paper is organized as follows: the next section describes the scope of application and the objectives taken into account in the approach; Section 3 discusses related work, proposed in the literature; Section 4 contains a complete description of the materials and methods used during the experiments; Section 5 details the recommended techniques to write efficient code; the experimental results are presented and discussed in Section 6, whereas current limitations and future work are addressed in Section 7. Finally, Section 8 concludes and summarizes the contributions of the paper.

## 2. Scope and Objectives

Several low-level optimization techniques are provided by compilers, more widespread than manual coding techniques, so that source code is compiled into a set of machine instructions to decrease the code size, use less memory, take less time to run, or perform fewer input/output operations. Their main objective is to achieve these advantages while preserving the semantics of the program, so that the algorithm remains unchanged.

Programming skills still have a role to play in this regard, given that the programmer has a large impact on the efficiency of the code written. This proposal aims to demonstrate that, in some cases, compilers such as GCC still do not achieve the same improvements that a programmer achieves when applying optimization techniques manually.

Additionally, this approach aims to provide useful guidelines for developers when writing efficient programs for IoT devices, so that they can achieve significant improvements with minimal effort by modifying the code. For a non-expert programmer, it can be difficult to know how to optimize a particular fragment of code so that it executes more rapidly, beyond the use of profilers and analysis tools to detect bottlenecks, or parts where the program spends most of its execution times. In this sense, it is important to highlight that an inefficient piece of code can waste a significant amount of time in the long term, when repeated millions of times. Thus, it is intended that programmers of Raspberry Pi and other ARM-based SBCs be aware of the important impact that even small and simple portions of code can have on their applications, especially when it comes to those that run continually.

Speeding up the executions may also lead to the achievement of higher energy efficiencies since, although energy consumption also depends on a power variable, reducing execution times implies a reduction in energy consumption when this power is constant over time. Assuming this, several works have demonstrated that energy usage and time are directly related [14], especially with regards to C and C++ languages [15,16], concluding that it is best to produce code that runs at full-throttle, completing as quickly as possible [17]. However, this cannot be considered a general rule, as it cannot be assumed that a decrease in execution time always results in an energy decrease.

The present approach is focused on C and C++, two of the most commonly used languages. The use of interpreted languages such as Java and Python was ruled out because they need to be translated during runtime, whereas for compiled languages the translation overhead is incurred just once, when compiling the source, and therefore (depending on the application) better execution times can be achieved [15]. In this sense, Fortran would also have been a good choice, but it is rarely used at present.

Regarding profilers and analysis tools, they can be an important tool for improving codes, but they are focused on detecting bottlenecks and measuring the execution times of each code fragment, rather than improving the implementation of the whole program by writing efficient code directly. As such, they are beyond the scope of this proposal. In addition, the use of profilers has been ruled out for our proposal because, although they are carefully designed to be as inexpensive as possible, they tend to introduce significant latency and overheads, influencing the runtime, which would be unacceptable for taking measurements.

It is also important to highlight that Raspberry Pi devices were chosen instead of other sensor nodes or smart objects, because of their ability to compute IoT data (within a reasonable time when the complexity of the problem increases) rather than sending it to a central location for processing. These types of devices are more suitable to demonstrate the important impact that the proposed techniques have on execution times, since they support a much greater computational load. Nevertheless, even if single-board microcontrollers are excluded, there are a large number of SBCs that could have been used to perform the experiments. It was decided to focus on Raspberry Pi devices because they stand out from the rest, due to the computing power they offer per watt, better cost/performance ratio, and especially as they have been used in a large number of representative and heterogeneous research projects (as mentioned above). In addition, after analyzing other SBCs, it was concluded that ARM architectures predominate widely in most devices. Thus, the results obtained with the Raspberry Pi devices can also be extrapolated to them.

Finally, higher efficiencies can be achieved thanks to parallelism and should be addressed in future works. In fact, a transcompiler for the automatic parallelization of sequential codes has been developed [18] to train users who are new to parallel programming. It is hoped that in the future, an extension of the current proposal can be applied to the development of this transcompiler to help resolve the challenges that the Internet of Things is currently facing, especially in terms of runtime and energy consumption.

## 3. Related Work

There are few proposals aimed at writing efficient code for IoT devices. In addition, most programmers have never been taught to consider efficiency when writing code.

As far as optimizing C and C++ codes is concerned, a proposal focused on the impact of compiler optimization on the efficiency of several well-known programs is presented in ref. [16]. Fog provides an extensive reference guide on optimizing C++ for Windows, Linux and Mac platforms, using CPU clock cycles rather than seconds or microseconds as time measure, based on the x86 family of microprocessors from Intel, AMD and VIA, including the 64-bit versions, in ref. [19]. In ref. [20], a complete chapter dedicated to efficient C programming (on ARM architectures in general, but without time estimates) can be found. A platform and independent compiler report on C++ performance is presented in ref. [21]. It gives the reader a model of time and space overheads implied by the use of various C++ language and library features, presents techniques for using C++ in applications where performance matters, and shows techniques for implementing C++ Standard language and library facilities to yield efficient code. In ref. [22] a significant amount of running examples are provided to demonstrate how to apply performance-tuning principles to improve existing C++ code, also offering timed results on an i7 processor (Intel Corporation, Santa Clara, CA, USA) with a Visual Studio 2010 compiler (Microsoft Corporation, Redmond, WA, USA). A system to optimize applying dead code and common subexpression eliminations is presented in ref. [23], whereas some methods for dead code elimination and inlining techniques in C/C++ language are described in ref. [24]. A complete set of compiler-based code-improvement techniques mostly focused on C code is presented in ref. [25], describing analytical techniques and specific data flow problems and discussing transformations to enhance the running times of programs on uniprocessor machines. A wide range of general guidelines and techniques used to optimize C code can be found in refs. [26,27,28].

In addition, recent proposals have focused on code refactoring techniques [29,30,31] and restructuring codes without changing external behavior, but they only cover a limited number of techniques, and do not contain enough detail to help developers in writing more efficient code.

Nevertheless, there is a lack of approaches that analyze the advantages of writing efficient code for RPi devices. Thus, the current proposal provides a set of tests which were developed exclusively for the measurement and analysis of twenty-five different techniques to reduce execution times, aimed at demonstrating the importance of applying certain efficient strategies when developing C/C++ codes for this type of device. These techniques were chosen because they are the most representative of those existing in the literature and so can be widely applied in an extensive range of programming routines and tasks, significantly impacting application runtime. In addition, they are easy to use for beginners as well as advanced programmers.

## 4. Materials and Methods

The experiments were developed on three types of Raspberry Pi devices: second-generation model B, third-generation model B and third-generation model B+, whose characteristics are shown in Table 1 [3]:

RPi devices can be powered in several ways: using a 5 V micro USB mains adaptor with 1200 mA power; using a USB-based portable battery compatible with smartphones; using the Mobile Pi Power (MoPi) (a power regulator that offers multiple inputs such as solar cells, car power sockets, or standard batteries, with the ability to swap power supplies without interruption); or using a battery box with six or more AA batteries [32]. For this proposal, the power adapter was chosen for its stability and reliability when it comes to maintaining the power.

All the experiments were conducted with an ambient temperature of 22 ${}^{\circ }$C, reaching the RPi’s maximum CPU temperature of 60 ${}^{\circ }$C. In addition, automatic pauses of 3 min were scheduled between each experiment to avoid possible overheating problems during measurements.

Raspbian, a port of the Linux Debian distribution optimized for the ARM instruction set, was used as the operating system, as recommended by the Raspberry Foundation [3]. Therefore, every Raspberry Pi used for the experiments had Raspbian Stretch 4.14 (June 2018 released), with all its packages updated on 1 October 2018. The operating system and all the files were stored in SanDisk Ultra microSDHC class 10 memory cards.

As far as compilers are concerned, all the code was compiled using GCC 6.3.0 version (May 2017 released) contained in the last stable version of GCC in the Raspbian6.3.0−18+rpi1+deb9u1 package. In order to analyze the improvements obtained through the automatic optimization, there were four levels of optimization available [13]:

- O0: without optimization for execution time. The objective of the compiler is to reduce the cost of compilation and to allow debugging to produce the expected results. This is the default. The other levels of optimization increase compilation time and the ability to debug the program may disappear.
- O1: optimization for code size and execution time.
- O2: the compiler performs nearly all supported optimizations that do not involve a space–speed tradeoff. This option increases both compilation time and the performance of the generated code.
- O3: maximum level of optimization (at the expense of compilation time, and possibly the ability to debug the program).

The details of all the optimization flags can be found in ref. [13], but it is also possible to obtain the exact set of optimizations that are enabled at each level by means of invoking GCC with −*Q* - -help =optimizers flags.

To carry out the experiments, and in order to improve the accuracy of the measurements, each test was repeated with at least one hundred million iterations, so that the runtime of the analyzed code grew to a measurable value and so it was also possible to discard the test function invocation cost. In addition, the end result was the average of ten different measurements in order to obtain ten execution time samples, reducing the impact of cold starts and cache effects. In this way, random variations were canceled out and the state of the caches tended to converge on a single value, avoiding outliers. The general pseudocode followed in all the experiments is shown in Algorithm 1.

**Algorithm 1** Time measurement.
1:
**procedure**
2:      initialization3:      **for** i←0,9 **do**4:            starttime5:            **for** j←0,100000000 **do**6:                 executetest7:           **end for**8:            gettime9:      **end for**10:     returnexecutionstatistics11:
**end procedure**



To ensure the accuracy of the tests, especially as far as the automatic optimization of the compiler is concerned, each test code was enclosed in a single function with an OPTIMIZE attribute in its declaration whose content was defined through preprocessor directives, as shown in Listing 1. In this way, to compile a program with, for instance, the maximum level of optimization, the compilation command should be invoked as follows: *g*++ source.c -*o* output -Dlevel=‘O3’.

Regarding the time measurement, the stopwatch class defined in ref. [22] was followed to time the tests, with the objective of obtaining meaningful results on performance measurement (see Listing 2). It consisted of a timer that provided functions to access a tick counter, using the chrono library, available from C++ version 11 and later, with the benefit of being portable to other operating systems. This option was chosen because of its resolution of around 10 milliseconds and its lack of overhead while running, so that the only latency laid in the system call to get the current time, which was not significant when timing tasks of tens of milliseconds or more [22].   

Listing 1 **Preprocessors directives.**


#**ifdef** level
#**define** OPTIMIZE __attribute__((optimize(level)))
#**else**
#**define** OPTIMIZE
#**endif**

Listing 2 **Stopwatch class used for the measurements.**


#**include** <chrono>
**using namespace** std::chrono;
 
**class** Watchtime{
std::chrono::system_clock::time_point m_start;
system_clock::duration diff;
**public**:
**void** startTime();
**unsigned** getTime();
};
 
**void** Watchtime::startTime(){
system_clock::time_point::min();                        // *clears the timer*
Watchtime::m_start = std::chrono::system_clock::now();  // *start the timer*
}
**unsigned** Watchtime::getTime(){
Watchtime::diff = system_clock::now() - m_start;
**return** (**unsigned**)(duration_cast<milliseconds>(diff).count());
}

The measurement procedure called the startTime function of the stopwatch class so that it started to count until it was stopped to get the elapsed time. After this, the result (difference between final and initial times) was converted to milliseconds. This measurement process was repeated ten times for each test, as mentioned above, to balance variations in the runtime.

With the objective of avoiding variations due to background tasks and context switching during the tests, the experiments started their executions by calls made through Secure Shell (SSH) network protocol, and only required services run, while unwanted services on Raspbian were reduced as much as possible before performing the experiments. Therefore, lightdmdesktop service and bluetooth and wireless drives were disabled, together with daemons such as avahi or triggerhappy.

## 5. Techniques to Write Efficient Code

This section provides a brief introduction to the techniques to write efficient code, which were selected from the existing literature [19,20,21,22,23,24,25,26,27,28]. All of them are focused on transformations aimed at decreasing the running time of the programs. Section 6 details, in more depth, those that have proven to be especially efficient when executing code in RPi devices.

Following Algorithm 1 presented above, two specific tests were developed for each technique: one test with a standard code, and a second with the result of applying the corresponding efficient technique on the first one. To get their associated runtimes (as discussed above), ten measurements were performed, and the tests were repeated one hundred million times for each measurement.

It is important to highlight the fact that in many cases, speeding up programs supposes code size increments that affect their readability. In addition, although all the experiments were based on C++ code, they apply equally to C. Another important point is the infinite possible variations in the experiments to analyze each technique. The main objective of these tests was to demonstrate that the proposed alternatives reach significant improvements in the runtime, even improving the automatic optimizations offered by the compiler when the techniques are not applied. Thus, we intend to demonstrate that it is necessary to take them into account when writing highly efficient code.

The source codes of all the proposed tests can be consulted in the Supplementary Materials. Next, all the techniques considered for their development are introduced.

T1 ***Bit fields***: a bit field is a data structure to hold a sequence of a certain number of bits with the objective of improving the use of memory space—for instance, to avoid the use of structures which require 8 bits of memory space, when only 2 of them are used. This is common with true/false variables. However, bit fields are usually accessed using pointers, which leads to aliasing problems. Consequently, in terms of speed, it is recommended to use an integer and, in any case, applying enum or #define masks to divide it. The experiment illustrates this problem, comparing the individual access to each of the four bits of a structure versus the use of a single integer.
T2 ***Boolean return***: returning several boolean variables at the end of a function is not a recommended practice, since each one of these returns implies using a whole register by itself. Instead, the conversion of multiple bools into a single unsignedint is advised, with the help of associated flag definitions. In this way, several checks can be made thanks to a single logical operation.
T3 ***Cascaded function calls***: as far as possible, cascaded function calls that return pointers or references should be avoided. For instance, in this experiment, the result of the call to the getData function, which is repeated *N* times, can be replaced by a single call by storing the reference to data and using it within the loop. In this case the programmer, unlike the compiler, can know that getData always returns the same reference.
T4 ***Row-major accessing***: languages such as Fortran or MATLAB support two-dimensional arrays stored in a contiguous by column-major order, whereas row-major is used in C and C++. This means that the element x+1 is stored right next to *x*. Thus, it is recommended to increment the leftmost index first when stepping through two-dimensional arrays, with the objective of achieving a higher rate of cache hits when accessing them. Both of these ways to traverse a two-dimensional array were used in the experiment.
T5 ***Constructor initialization lists***: regarding the constructors, the use of initialization lists is recommended when setting the initial values of the variables. Otherwise, assignments within the body imply that the default constructors of the variables are always invoked, with the consequent overhead. For instance, in the experiment the string constructor is called first for two attributes, then the assignment operator of string is called inside the body of a class constructor to assign the values. Afterwards, the destructor of string is called when the two attributes got out of scope. This code is compared to another one in which, in a class, the arguments in the initialization list are used to copy the variables directly.
T6 ***Common subexpression elimination***: identical expressions that are computed repeatedly, without changing the values of their operands, can be replaced with single variables that hold the computed values. The experiment analyses the effect of storing an addition operation with an integer and a square root in a local variable, to use in the rest of the code.
T7 ***Mapping structures***: the use of tables when mapping values to constants, changing the checklist of each of the possible cases (with nested if statements) by a loop that traverses the table looking for the right constant is recommended.
T8 ***Dead code elimination***: this is one of the best known and widely applied optimizations by compilers. When an instruction is totally unreachable or does not influence the result (e.g., in the case of variables that are never used), it can be eliminated. The experiment analyses the impact of two never-used variables assignments and an unreachable line.
T9 ***Exception handling***: this is used with the objective of changing the normal flow of an execution when anomalous or exceptional conditions occur. In this way, exceptions are necessary to deal with unexpected errors. In fact, it should be noted that all the containers in the C++ standard library use new expressions that throw exceptions [22]. However, in some cases it is possible to significantly improve performance when they are used within a loop, since they can be replaced by continue or break statements, as applied in this experiment.
T10 ***Global variables within loops***: regarding the use of assignments to global variables within loops, it is recommended to move these allocations out of the loops by computing the corresponding operations in local variables and not performing the assignments in the global variables until the end of the loop is reached. The benefits of using this technique are analyzed in the experiment.
T11 ***Function inlining***: it is an effective method to completely remove the overhead caused each time a function is called. In fact, it is reported by some researchers as the most powerful code optimization [22]. Besides, this technique is preferred over the use of #define macros because the latter does not check the types of function [20]. It consists of replacing a call, inserting the code for the function, instead of calling it and thus removing call and return statements. A simple example is used in the experiment, where an addition function is expanded inline inside a subtraction one.
T12 ***Global variables***: global variables are defined outside all functions, so their scope is the whole program. Therefore, their use is not recommended in order to avoid functions interfering with each other as little as possible, since global values can be changed by any function and may cause programming problems later. In addition, global variables are not allocated to registers, so they should not be used within critical loops in order to avoid unnecessary overhead. Thus, regarding runtime, if a function uses global variables heavily, it is also beneficial to copy their values to local variables and use these instead to improve access times, as discussed in the experiment.
T13 ***Constants inside loops***: as far as possible, continuous access to constants within loops should be avoided. In many cases, however, this often leads to poor readability.
T14 ***Initialization versus assignment***: it is recommended to initialize variables directly, instead of using a subsequent assignment, and so avoid the unnecessary assignment of the instantiation. The experiment evaluates the benefit of this technique when using complex numbers.
T15 ***Division by a power-of-two denominator***: this technique consists of changing division expressions with power-of-two denominators by shift expressions, as shown in the experiment.
T16 ***Multiplication by a power-of-two factor***: this technique, similar to the previous one, consists of changing multiplication expressions with power-of-two factors by shift expressions.
T17 ***Integer versus character***: the use of integers is preferred over characters when performing arithmetic operations because C and C++ convert char values to integers before operating, later converting the result back to char again. The experiments analyze the effects of using characters or integers to make a sum.
T18 ***Loop count down***: this technique can be applied when the order of the loop counter is not determinative. It consists of traversing the loop in the opposite direction to take advantage of the fact that it is quicker to process “*i*- -” as the loop condition. Instead, traversing the loop forward (i=0;i<100 condition) means more steps: subtract *i* to 100, evaluate if the result is zero, if not, increment the iterator and continue, which may result in a significant time difference. This deviation is increased when in addition the loop is controlled by a while statement, as analyzed in the experiment. Another important point that must be taken into account is the reduction of the cost of the loop termination clause, given the extensive number of times that it can be evaluated.
T19 ***Loop unrolling***: there is the possibility of unrolling small loops to improve runtime, reducing the number of iterations and repeating the loop body several times. However, loop unrolling has an important disadvantage (i.e., code size is increased), so it should be applied carefully. Therefore, to avoid decreasing the performance of the cache, only unrolling loops which really affect the final runtime is recommended.
T20 ***Passing structures by reference instead of value***: structures should be passed by reference on function calls whenever possible in order to avoid the overhead associated with the complete copy of each structure (including constructor and destructor) when passed by value. Since reference arguments can modify the original instance (unlike value arguments), it is recommended that pointers to structures be declared as constants when their pointed values are not going to change. The experiment consists of a class with two string members and a data structure containing one integer attribute which is passed by reference in a first test and by value in a second one.
T21 ***Pointer aliasing***: this refers to the case where the same address is pointed to by two or more pointers. Thus, writing to one will affect the reading from the other. Due to this possibility, the compiler is prevented from applying certain optimizations, affecting efficiency. The experiment consists of a common subexpression elimination (aforementioned in T6) involving memory accesses through pointer, which cannot be automatically optimized by the compiler due to aliasing. There are four addition operations, which add the value pointed to by the same pointer to integer to the values stored in four different addresses (also pointers to integer).
T22 ***Chains of pointers***: this technique is directly related to the previous one, since it consists of an optimizing chain of pointers (a pointer which points to another pointer, and so on). The experiment analyzes the effect of caching a pointer to an object in a local variable, to then access the rest of the pointers instead of using the entire chain every time.
T23 ***Pre-increment versus post-increment***: the use of pre-increment operators is preferred with data types whose classes overload pre-increment and post-increment operators. While the post-increment operator needs to create a previous copy of the object, the pre-increment avoids it.
T24 ***Linear search***: generally, linear search through a list implies two main comparisons—one to control the loop iterations, and another to determine if the current key is the desired one. The performance can be improved with a single comparison if a while statement is used (while(list[i]!=searched)). To avoid problems when the searched value is not contained in the list, the size of the array should be increased and the desired value inserted at the end. In this way, the algorithm can know if the value was contained in the list by simply checking that the found index does not correspond to the last position.
T25 ***Invariant IF statements within loops***: regarding loops, another technique that can be applied consists of transforming one loop into two when it contains a loop-invariant if statement. For this, it is necessary to take the conditional expression out of the loop, so that one identical loop is traversed within each condition of the if.

## 6. Experimental Results and Discussion

The runtime results of the tests related to the aforementioned techniques are shown in Table 2, Table 3 and Table 4, organized by the device in which the experiments are executed (RPi 2 model B, RPi 3 model B and RPi 3 model B+).

As described in Section 4, each test was introduced into a loop (to be repeated to at least one hundred million iterations), and in turn this loop was executed ten times, in order to obtain the average result of those ten measurements. This initial runtime result, given in milliseconds, was divided by the number of iterations (getting the runtime of a single execution) and converted to nanoseconds for readability. The standard deviation was also calculated in order to quantify the dispersion of the measurements.

As mentioned above, two specific tests were developed for each technique: the first one with standard code, and the second with the result of applying the corresponding efficient technique on the first one (to decrease its runtime). In this way, the tables show the execution times in nanoseconds of both tests (standard code and efficient code) for each technique, applying and not applying the automatic optimization offered by the compiler—hat is, with optimization level three and without optimization (optimization level zero), respectively.

In addition, the percentage of improvement between the execution of the standard code and the efficient one is also shown, to facilitate the analysis of the results.

The standard deviations obtained were very small (with an average deviation of 0.31%), thereby indicating that the measurements tended to be close to the means given in the tables. The standard deviations were not included in the tables, but they can be consulted in the Supplementary Materials (Spreadsheet S2: complete experimental results).

Note that although the runtime differences between a standard code and its optimized version may seem insignificant, they refer to a single execution, so they become quite important when they are executed iteratively in loops with long execution time. This is especially important within programs that are running 24/7 throughout the year, something very common in IoT and these types of devices.

As expected, the execution times of the codes compiled with the maximum level of optimization (3) were significantly lower (applying and without applying the techniques). However, it was also important to analyze the effects of writing efficient code without the optimization offered by the compiler, because that way the ability to debug the programs remains unaltered and the expected results can be obtained. Therefore, in cases in which the debugging process takes a lot of time, employing techniques to write efficient code can imply substantial improvements. Nevertheless, results related to other optimization levels (1 and 2) were not included in the tables, as they were not particularly representative for the objectives of this article because they provided values that did not improve results. However, they can be consulted in the Supplementary Materials.

**Figure 1:**
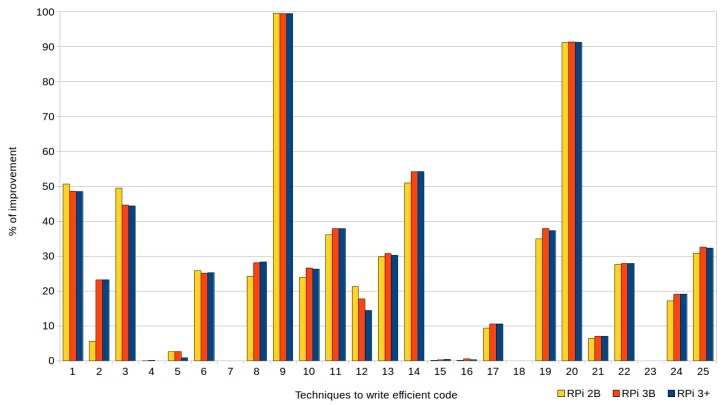
Percentages of improvement in the runtime achieved by writing efficient code (without compiler optimization).

**Figure 2:**
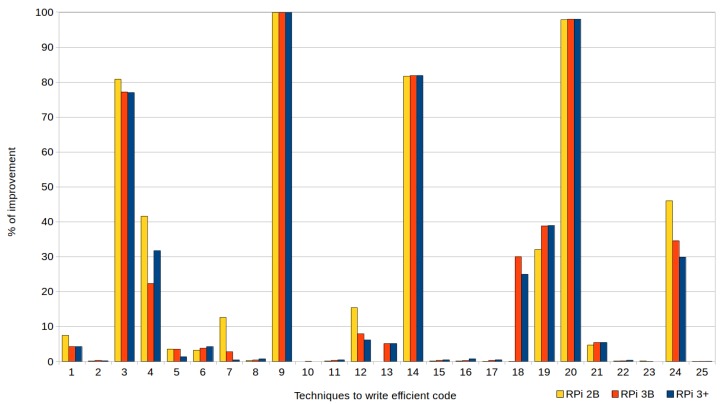
Percentages of improvement in the runtime achieved by writing efficient code (with compiler optimization level 3).

Figure 1 and Figure 2, corresponding to the previous tables, are provided to facilitate understanding of the data. They show the percentages of improvement in the execution times when efficient codes are used, with respect to their standard versions, in the three types of devices (RPi 2 model B, RPi 3 model B and RPi model B+). In the case of the executions represented by Figure 1, the automatic optimizations offered by the compiler were not used (i.e., the optimization level was set to zero (0) when compiling the tests), whereas Figure 2 corresponds to the tests compiled with the highest level of optimization (3).

As shown in the tables and both figures, although the more advanced the Raspberry Pi model was the shorter its execution times were. There were no significant differences in the percentages of improvement when applying the techniques in one model or another, except for the techniques that used mapping structures (T7) and avoided global variables (T12), since RPi 2B achieved (with optimization level 3) improvements of 10% compared to the other devices (this does not mean that their execution times were lower, but that the impact of applying the technique was greater).

Reductions of up to 99.53% could be achieved using these efficient techniques, as shown in both the figures and the tables. Particularly noteworthy are the results (discussed below) obtained with the exception handling technique (T9) and passing structures by reference instead of by value (T20), to be expected given that both are widely known.

Regarding the writing of efficient codes without compiler optimization (see Figure 1), improvements were achieved in most cases, except in seven, where the efficient codes reached almost the same runtimes as the standard codes. The effectiveness of the following techniques could not be demonstrated, despite having made substantial efforts to prove their usefulness: row-major accessing (T4); constructor initialization lists (T5); mapping structures (T7); division by a power-of-two denominator (T15); multiplication by a power-of-two factor (T16); loop count down (T18); and pre-increment versus post-increment (T23). In fact, if these techniques were discarded, the others achieved an average improvement of 36.20%. In this way, 16 techniques demonstrated their efficiency to write efficient code when compiler optimization was set to default (i.e., with level zero).

In the case of writing efficient codes combined with the application of optimization level 3, the compiler successfully resolved most of the cases, as shown in Figure 2. However, as can be observed, it is still recommended to write efficient code manually in relation to the following eight techniques, whose average percentage of improvement in the runtime achieved when applying them was approximately 60%: cascaded function calls (T3); row-major accessing (T4); exception handling (T9); initialization versus assignment (T14); loop count down (T18); loop unrolling (T19); passing structures by reference (T20); and linear search (T24). Next, they were analyzed in greater depth, evaluating the factors which conditioned their improvement but focusing only on the RPi 3B+, as this was the device which obtained the best execution times (as expected).

### 6.1. Cascaded Function Calls

In this technique, the standard code consists of a loop that, in each iteration, checks the content of an integer variable calling a function through a pointer, which returns the value of the variable. In the efficient code, the value of the variable is stored in an auxiliary variable, so that the function is only called once (outside the loop), and it is the auxiliary variable which is checked in all iterations. This technique can be applied in cases where the programmer (unlike the compiler) knows that the value of the variable does not change during the execution of the loop. The code of both tests can be shown in Listing 3. Writing the efficient alternative by hand results in an improvement of over 70%, in just 15 iterations of the loop (N=15).

Listing 3 **Cascaded function calls. Standard and efficient tests developed.**

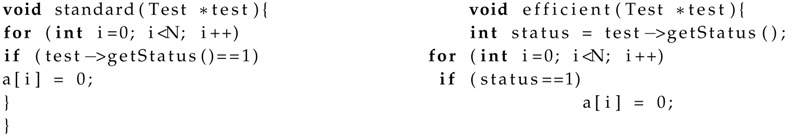

**Figure 3:**
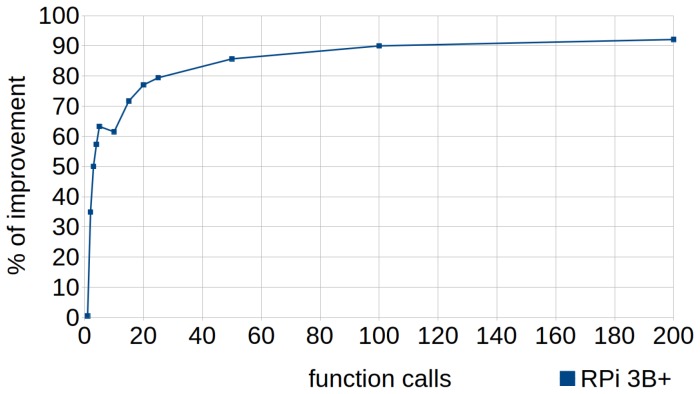
Cascaded function calls. Percentage of improvement in the runtime according to the number of calls to the function (number of iterations of the loop) in RPi 3B+.

Figure 3 shows how the percentage of improvement varied according to the number of calls to the function—that is, the number of iterations of the loop. A single call to the function achieved a small improvement of 0.49%, whereas with two calls it already reached 35%. From 100 to more calls, the percentage grew very slowly, stabilizing around an improvement of 90%. Therefore, it is advisable to apply this technique whenever possible under the above conditions.

### 6.2. Row-Major Accessing

This technique is based on the recommendation of always traversing the data in the order it was stored, incrementing the leftmost index first when stepping through two-dimensional arrays. The objective is to achieve higher rates of cache hits when accessing them, since C/C++ stores them in a contiguous by row-major order—that is, row-by-row (values are written putting the first row in contiguous memory, then the second row right after it, and so on). Thus, in the test developed for this technique, the standard code traverses the array iterating over each column before going to the next one (processing the columns in the outer loop and the rows in the inner loop), whereas the efficient code consists of traversing using row-major iteration (see Listing 4).

**Figure 4:**
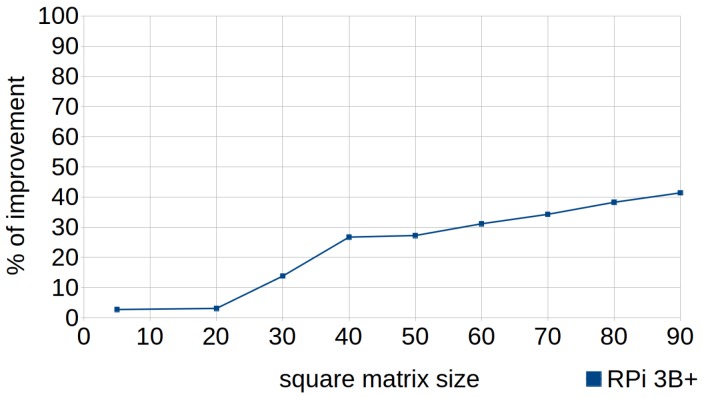
Row-major accessing. Percentage of improvement in the runtime according to the matrix size in RPi 3B+.

Figure 4 shows how the percentage of improvement obtained by applying this technique grew almost linearly depending on the size of the vector (larger size, greater percentage of improvement), due to the increase in the work of the cache and the reduction of efficiency when column-major iteration was used.

Listing 4 **Row-major accessing. Standard and efficient tests developed.**

**int** standard(){                              **int** efficient(){
**for** (**int** j=0; j<N; j++)                       **for** (**int** i=0; i<N; i++)
**for** (**int** i=0; i<N; i++)                       **for** (**int** j=0; j<N; j++)
array[i][j] = 0;                              array[i][j] = 0;
}                                            }

Nevertheless, this test was focused on arrays with the same number of rows and columns (representing square matrices). Hence, it is noteworthy to mention that the improvement would be influenced by the size of both dimensions. For instance, when traversing a narrow matrix (with few columns) by column-major order, consecutive rows may be found in the cache, and therefore the results obtained by executing the standard code would improve substantially. Thus, analysis of the diverse conditions depending on variations in the size of both dimensions of the arrays will be undertaken in future work.

### 6.3. Exceptions

This technique is based on the fact that in some cases the use of exceptions within loops can be avoided by using continue or break statements. The degree of improvement depends directly on the number of exceptions that are thrown. Note that, in the experiment, the runtime of the standard test referred to the throw of 100 simple exceptions, without any type of content except its declaration (classmyexception:publicexception{}myex;), achieving improvements of 99% when using the efficient version (see Listing 5). This time penalty was mainly due to the call to the exception handler, which includes passing a parameter as argument. The type of this argument should be checked against the type of parameter specified by the handler. If they match, the exception is caught and the control is transferred to the handler. Thus, although exceptions are necessary to support error handling, this technique is recommended when exceptions are within loops and there is a relatively high probability of occurrence, together with the possibility of being managed without throwing them.

Listing 5 **Exceptions. Standard and efficient tests developed.**

**int** num = 100;                               **int** num = 100;
**for** (**int** i=0; i<1; i++){                     **for** (**int** i=0; i<1; i++){
**try**{                                         **if** (num != 100) {
**if** (num == 100) {                          **continue**;
**throw** myex;                        }
}                                    }
} **catch** (exception& e){                   **return** 0;
}                                      }
}
**return** 0;
}

### 6.4. Initialization versus Assignment

Regarding this technique, the tests use complex double numbers, declared as a variable whose real and imaginary parts are both of type double. In the efficient code, the test is performed initializing the variable directly, whereas in the standard code, a subsequent assignment is used, as shown in Listing 6. In this case, the percentage of improvement achieved by writing efficient code reached 80% with respect to the use of the standard code, thanks to avoiding the unnecessary assignment of the instantiation. In general, it is recommended to use this technique with any type of data.

Listing 6 **Initialization versus assignment. Standard and efficient tests developed.**

**void** standard() {                            **void** efficient() {
std::complex<**double**> mycomplex;              std::complex<**double**> mycomplex(3.14);
mycomplex = (3.14);                       }
}

### 6.5. Loop Count Down

**Figure 5:**
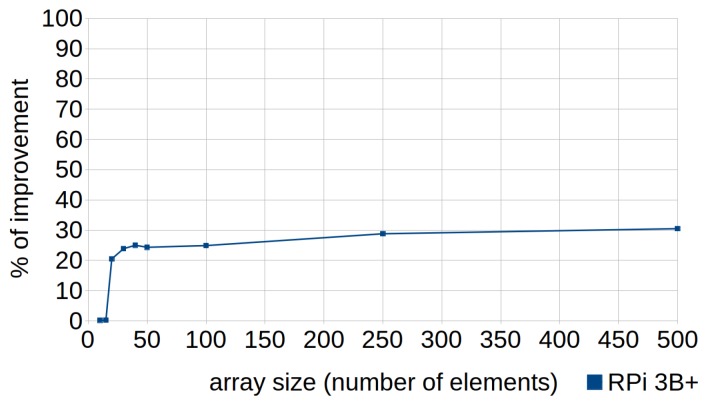
Loop count down. Percentages of improvement in the runtime according to the array size (number of elements) in RPi 3B+.

This technique can be applied in cases where the order of the loop counter is not determinative. The standard code traverses the loop forward while the efficient code consists of traversing it in the opposite direction, as shown in Listing 7. Its use resulted in improvements from 20% to 30% with array sizes greater than 20 elements (see Figure 5). This is because it is quicker to process “*i* - -” as the while condition when traversing it in the opposite direction. As mentioned in Section 5, traversing the loop forward implies more steps: subtract *i* to *N*, evaluate if the result is zero; if not, increment the iterator and continue. In this sense, reductions in the cost of the loop termination clauses would also mean significant improvements, given the extensive number of times that they can be evaluated.

Listing 7 **Loop count down. Standard and efficient tests developed.**

**void** standard(){                             **void** efficient() {
**for** (**int** i=0; i<N; i++) {                      **int** i = N+1;
a[i]=i;                                    **while** (--i) {
}                                                   a[i] = i;
}                                                }
}

### 6.6. Loop Unrolling

**Figure 6:**
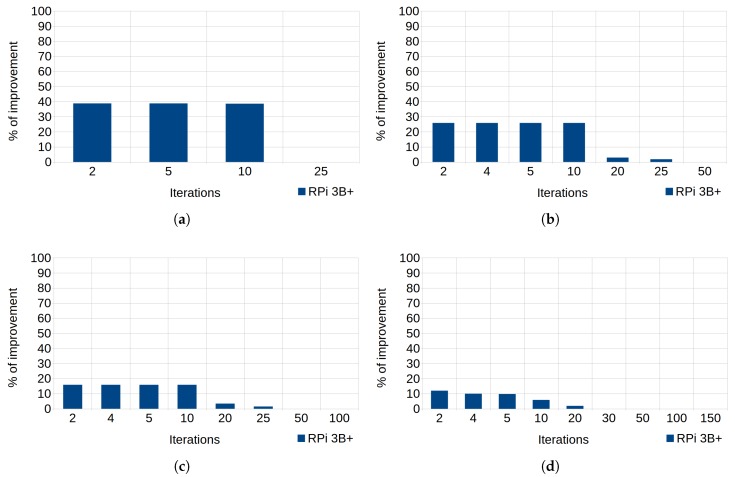
Loop unrolling in RPi 3B+. Percentages of improvement in the runtime according to the number of iterations. (**a**) Array size of 50 elements. (**b**) Array size of 100 elements. (**c**) Array size of 200 elements. (**d**) Array size of 300 elements.

In the case of the loop unrolling technique, the tests consist of initializing an integer array so that the standard code traverses the loop forward and performs an assignment (initialization to zero) in each iteration (see Listing 8), whereas the efficient code unrolls the loop and performs five assignment operations in each iteration, consequently increasing the code size while reducing the number of iterations. Figure 6 helps in the analysis of the execution results of these tests.

Listing 8 **Loop unrolling. Standard and efficient tests developed.**

**void** standard(){                             **void** efficient(){
**int** i;                                       **int** i;
**for** (i=0; i<N; i++){                         **for** (i=0; i<N; i+=5){
array[i] = 0;                                array[i]   = 0;
}                                              array[i+1] = 0;
}                                                  array[i+2] = 0;
array[i+3] = 0;
array[i+4] = 0;
}
}

In Figure 2, the percentage of improvement reached 39% with an array of 50 elements (N=50). However, it is necessary to understand the factors that affect this technique, since it depends on both the size of the vector and the number of unrolled operations. Figure 6a–d matches arrays of 50, 100, 200, and 300 elements, respectively, and represent the percentages of improvement according to the number of iterations. Taking as example the array of 100 elements, 50 assignment operations are needed to initialize the entire vector with only two iterations of the loop, 25 assignments for 4 iterations, and so on, until reaching 50 iterations (which only need two assignment operations). As can be observed, the smaller the vector, the greater the improvement achieved using this technique. In fact, the improvement was not remarkable when the unrolling was used to initialize arrays of 200 or more elements.

As can be observed, regarding the array of 50 elements (see Figure 6a), there was an improvement of around 40% when the number of iterations was smaller than 10 (there were more than 10 assignment operations unrolled). Thus, when the number of iterations grew (exceeding 20 iterations) the improvement disappeared. The same applies to the other three arrays.

### 6.7. Passing Structures by Reference Instead of Value

In this technique, the standard test passes a structure by value and directly accesses the value of an integer variable (see Listing 9), whereas in the efficient version the structure is passed by reference and the content of the variable is accessed through a pointer. In both cases, the structure consists of a class with two string variables, and a sub-structure with an array of 10 elements and an integer variable. In this way, passing the structure by reference resulted in an improvement of 98%. As mentioned in Section 5, this was due to the overhead associated to the complete copy of each structure (including constructor and destructor) when passed by value. In addition, since reference arguments can modify the original instance (unlike value arguments), it is recommended that pointers to structure be declared as constants when their pointed values are not going to change.

Listing 9 **Passing structures by reference. Standard and efficient tests developed.**

**typedefstruct** {**int** array[10]; **int** index;} Structure;
**class** Class {
**private**:
string    attribute_a ;
string    attribute_b;
Structure structure;
**public**:
Class(string attribute1, string attribute2, **int** i);
**int** getIndex();
};
Class::Class(string attribute1, string attribute2, **int** i) {
attribute_a = attribute1;
attribute_b = attribute2;
structure.index = i;
}
**int** Class::getIndex() {
**return** structure.index;
}
  
**int** standard(Class value){                   **int** efficient(Class *reference){
**return** value.getIndex();                    **return** reference->getIndex();
}                                            }

### 6.8. Linear Search

Although there are other search algorithms that achieve faster results, linear search is especially recommended in short lists or for users who are beginners in programming. This technique consists of finding a target number within an integer array (see Listing 10). The standard code is based on a linear search, so that each element is sequentially checked until a match is found (returning the index) or until all the elements have been searched (the target value does not match with any element and the function returns −1). Consequently, two comparisons are required: one to control the loop iterations, and another to determine if the current key is a value match. In the efficient code, a while statement is used to make a single comparison in each iteration. To achieve this, it is necessary to increase the size of the array and insert the target value in the last position so that if the found index does not match the last position, the value was contained in the array. In both tests, the arrays are initialized so that the searched number is in their last position.

**Figure 7:**
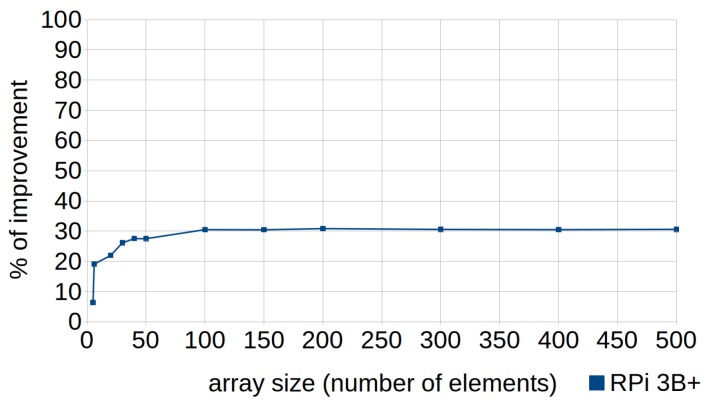
Linear search with for loops. Percentages of improvement in the runtime according to the array size (number of elements) in RPi 3B+.

The use of this technique resulted in improvements of between 20% and 30% with arrays of six or more elements, as shown in Figure 7.

Listing 10 **Linear search. Standard and efficient tests developed.**

**int** standard(**int** *list, **int** N, **int** search){    **int** efficient(**int** *list, **int** N, **int** search){
**int** i;                                       **int** i;
**for** (i = 0; i < N; i++)                      list[N] = search;
**if** (list[i] == search)                     i = 0;
**return** i;                                **while** (list[i] != search)
**return** -1;                                       i++;
}                                               **if** (i == N)
**return** -1;
}

## 7. Limitations and Future Work

The main objective of the experiments was to demonstrate that the proposed alternatives achieved significant improvements in the runtime, even improving the automatic optimizations offered by the compiler if the techniques were not applied. However, there are infinite possible variations which could have been applied and analyzed in each of the experiments, which were also conditioned to the programming language (as discussed in Section 2), the device, and its hardware architecture, as well as the workloads used.

Therefore, future work is expected to expand the analysis of the seven techniques that were proven to be essential regarding the improvement of runtimes in Raspberry Pis. This involves evaluating all those conditions that influence such improvements, including, for example, different data types or varied workloads, among other essential circumstances that should be taken into account in order to manually write efficient code. In the same way, the possibility of analyzing the effects of combining several techniques will also be taken into account. Moreover, modeling and simulation could also be crucial for properly evaluating the energy savings achieved when writing efficient code.

The analysis in other IoT devices, apart from Raspberry Pis, as well as the use of other compilers (and even other programming languages) will also be addressed in future work, to determine if these techniques can be applied to other types of IoT hardware, regardless of architecture. In addition, a software and hardware codesign has not been ruled out, since a joint specification, design, and synthesis of hardware and software IoT systems could increase the improvements already achieved.

In addition, it is evident that higher efficiencies can be achieved thanks to parallelism. Thus, it is hoped that in the future the current proposal can be extended and applied to help users who are new to parallel programming (as mentioned in Section 2), and thus help resolve the challenges that the Internet of Things currently faces.

## 8. Conclusions

This paper analyzed a broad set of techniques used to write efficient programming codes for Raspberry Pi devices. All of these techniques, which were selected from the existing literature, are focused on transformations aimed at decreasing execution times while preserving the semantics of the program, so that the algorithm remains unchanged. In addition, the proposal provides a set of tests that were developed to allow the measurement and analysis of the proposed efficient techniques.

We demonstrated that, in some cases, compilers such as GCC still do not achieve the same improvements that a programmer achieves when applying optimization techniques manually. Thus, the approach evaluated the execution times obtained through the manual application of these techniques, compared to the automatic optimizations offered by the compiler, which were insufficient when it came to obtaining the best efficiency.

Thus, 25 techniques were analyzed, of which 17 demonstrated their effectiveness when writing efficient codes without the optimization offered by the compiler. In the case of the application of these techniques combined with an optimization (level 3), the results show that there were eight cases that should be applied manually, given that the compiler demonstrated its inadequacy. These eight cases were analyzed in greater depth, evaluating the factors which condition the improvements in the execution times. In addition, the results obtained can also be extrapolated to other devices with similar ARM architectures.

In this way, it is intended to show that programmers of Raspberry Pi and other ARM-based SBCs should be aware of the important impact that even small and simple portions of code can have on their application’s runtime (which may also lead to a higher energy efficiency), and to apply the recommended techniques, especially when their programs are developed to run continually.

## Figures and Tables

**Table 1:** Raspberry Pi (RPi) comparison chart.

Model	RPi 2 B	RPi 3 B	RPi 3 B+
**SOC Type**	Broadcom BCM2836	Broadcom BCM2837	Broadcom BCM2837B0
**CPU Clock**	900 MHz Quad Core ARM Cortex-A7	1.2 GHz Quad Core ARM Cortex-A53	1.4 GHz Quad Core ARM Cortex-A53
**RAM**	1 GB	1 GB	1 GB
**GPU**	Broadcom VideoCore IV 1080p30	Broadcom VideoCore IV 1080p60	Broadcom VideoCore IV 1080p60
**USB Ports**	4	4	4
**Ethernet**	100 Mbit/s base Ethernet	100 Mbit/s base Ethernet	Gigabit Ethernet over USB 2.0 (maximum throughput 300 Mbps)
**Power over Ethernet**	No	No	Yes (requires separate PoE HAT)
**WiFi**	No	On Board WiFi 802.11n	On Board WiFi 802.11ac Dual Band 2.4 GHz & 5 GHz
**Bluetooth**	No	On Board Bluetooth 2.0/4.1	On Board Bluetooth 2.0/4.1/4.2 LS BLE
**Video Output**	HDMI 3.5 mm Composite DSI (for LCD)	HDMI 3.5 mm Composite DSI (for LCD)	HDMI 3.5 mm Composite DSI (for LCD)
**Audio Output**	I${}^{2}$ S HDMI 3.5 mm Composite	I${}^{2}$ S HDMI 3.5 mm Composite	I${}^{2}$ S HDMI 3.5mm Composite
**Camera Input**	15 Pin CSI	15 Pin CSI	15 Pin CSI
**GPIO Pins**	40	40	40
**Memory**	MicroSD	MicroSD	MicroSD

**Table 2:** Runtime results and percentages of improvement in Raspberry Pi 2B.

Technique		Runtime (without Optimization)			Runtime (Optimization Level 3)		
		Standard (ns)	Efficient (ns)	Improvement (%)	Standard (ns)	Efficient (ns)	Improvement (%)
1	Bit fields	85.71	42.28	50.67	31.25	28.92	7.46
2	Boolean return	40.63	38.35	5.61	20.32	20.30	0.10
3	Cascaded function calls	828.28	418.60	49.46	514.83	98.46	80.88
4	Row-major accessing	94,096.73	94,096.72	0.00	12,850.10	7502.61	41.61
5	Constructor initialization lists	1158.58	1128.70	2.58	1148.76	1107.63	3.58
6	Common subexpression elimination	74.50	55.29	25.79	36.13	34.97	3.21
7	Mapping structures	522.61	812.45	−55.46	505.80	442.10	12.59
8	Dead code elimination	32.47	24.61	24.21	16.82	16.78	0.24
9	Exception handling	9465.32	42.51	99.55	9406.65	10.06	99.89
10	Global variables within loops	558.62	425.40	23.85	87.27	87.29	−0.02
11	Function inlining	55.66	35.60	36.04	20.15	20.13	0.10
12	Global variables	1538.84	1211.68	21.26	697.21	589.75	15.41
13	Constants inside loops	504.34	353.86	29.84	224.77	228.08	−1.47
14	Initialization versus assignment	57.09	27.96	51.02	54.55	10.01	81.65
15	Division by a power-of-two denominator	35.83	35.79	0.11	20.16	20.13	0.15
16	Multiplication by a power-of-two factor	35.63	35.60	0.08	20.05	20.02	0.15
17	Integer versus character	60.45	54.82	9.31	20.14	20.13	0.05
18	Loop count down	1483.21	1824.74	−23.03	360.35	360.29	0.02
19	Loop unrolling	770.95	501.29	34.98	115.28	78.31	32.07
20	Passing structures by reference	509.51	44.75	91.22	474.79	10.06	97.88
21	Pointer aliasing	121.96	114.11	6.44	71.63	68.24	4.73
22	Chains of pointers	84.58	61.19	27.65	24.64	24.61	0.12
23	Pre-increment versus post-increment	2706.01	2707.06	−0.04	692.16	690.97	0.17
24	Linear search	2487.52	2060.83	17.15	700.99	378.30	46.03
25	Invariant IF statements within loops	2161.82	1496.00	30.80	156.92	156.88	0.03

**Table 3:** Runtime results and percentages of improvement in Raspberry Pi 3B.

Technique		Runtime (without Optimization)			Runtime (Optimization Level 3)		
		Standard (ns)	Efficient (ns)	Improvement (%)	Standard (ns)	Efficient (ns)	Improvement (%)
1	Bit fields	55.09	28.35	48.54	21.05	20.16	4.23
2	Boolean return	32.84	25.23	23.17	12.65	12.61	0.32
3	Cascaded function calls	532.65	295.25	44.57	309.11	70.57	77.17
4	Row-major accessing	61,209.77	61,139.89	0.11	6796.87	5282.00	22.29
5	Constructor initialization lists	766.16	746.05	2.62	757.83	731.68	3.45
6	Common subexpression elimination	50.05	37.52	25.03	26.20	25.22	3.74
7	Mapping structures	399.75	524.69	−31.25	381.80	371.36	2.73
8	Dead code elimination	21.03	15.12	28.10	10.96	10.92	0.36
9	Exception handling	5465.30	26.68	99.51	5460.63	6.67	99.88
10	Global variables within loops	382.89	281.08	26.59	74.11	74.07	0.05
11	Function inlining	37.88	23.54	37.86	12.54	12.50	0.32
12	Global variables	910.18	749.08	17.70	521.35	480.29	7.88
13	Constants inside loops	340.73	236.00	30.74	144.84	137.48	5.08
14	Initialization versus assignment	40.35	18.48	54.20	37.03	6.72	81.85
15	Division by a power-of-two denominator	23.41	23.35	0.26	12.55	12.51	0.32
16	Multiplication by a power-of-two factor	23.47	23.35	0.51	12.64	12.61	0.24
17	Integer versus character	40.07	35.86	10.51	12.64	12.60	0.32
18	Loop count down	1027.85	1292.02	−25.70	269.68	188.72	30.02
19	Loop unrolling	532.41	330.48	37.93	93.45	57.18	38.81
20	Passing structures by reference	348.64	30.02	91.39	332.00	6.72	97.98
21	Pointer aliasing	84.31	78.39	7.02	48.47	45.86	5.38
22	Chains of pointers	60.54	43.69	27.83	16.82	16.79	0.18
23	Pre-increment versus post-increment	1867.60	1868.37	−0.04	519.81	519.78	0.01
24	Linear search	1690.00	1368.81	19.01	435.44	284.90	34.57
25	Invariant IF statements within loops	1538.42	1038.22	32.51	123.54	123.50	0.03

**Table 4:** Runtimes results and percentages of improvement in Raspberry Pi 3B+.

Technique		Runtime (without Optimization)			Runtime (Optimization Level 3)		
		Standard (ns)	Efficient (ns)	Improvement (%)	Standard (ns)	Efficient (ns)	Improvement (%)
1	Bit fields	47.92	24.67	48.52	18.18	17.41	4.24
2	Boolean return	28.35	21.77	23.21	10.90	10.88	0.18
3	Cascaded function calls	460.35	256.04	44.38	267.48	61.58	76.98
4	Row-major accessing	53,434.46	53,584.19	−0.28	6764.78	4622.89	31.66
5	Constructor initialization lists	673.66	667.99	0.84	662.28	653.39	1.34
6	Common subexpression elimination	43.01	32.16	25.23	22.46	21.52	4.19
7	Mapping structures	338.54	460.12	−35.91	324.79	323.35	0.44
8	Dead code elimination	17.94	12.86	28.32	9.50	9.43	0.74
9	Exception handling	4988.09	23.59	99.53	5056.53	6.03	99.88
10	Global variables within loops	330.50	243.72	26.26	63.54	63.81	−0.42
11	Function inlining	32.21	20.01	37.88	10.93	10.88	0.46
12	Global variables	793.95	679.45	14.42	464.86	436.42	6.12
13	Constants inside loops	289.83	202.27	30.21	126.07	119.64	5.10
14	Initialization versus assignment	34.35	15.72	54.24	31.52	5.71	81.88
15	Division by a power-of-two denominator	20.08	20.01	0.35	10.93	10.88	0.46
16	Multiplication by a power-of-two factor	20.07	20.01	0.30	10.80	10.72	0.74
17	Integer versus character	34.88	31.20	10.55	10.77	10.72	0.46
18	Loop count down	889.60	1103.27	−24.02	240.11	180.32	24.90
19	Loop unrolling	456.63	286.43	37.27	79.51	48.60	38.88
20	Passing structures by reference	301.95	26.29	91.29	285.03	5.85	97.95
21	Pointer aliasing	72.21	67.18	6.97	41.57	39.31	5.44
22	Chains of pointers	51.51	37.16	27.86	14.34	14.29	0.35
23	Pre-increment versus post-increment	1611.73	1652.95	−2.56	469.88	486.87	−3.62
24	Linear search	1455.65	1177.63	19.10	386.03	270.78	29.86
25	Invariant IF statements within loops	1321.56	895.18	32.26	105.90	105.86	0.04

## Supplementary Materials

The following are available at http://www.mdpi.com/1424-8220/18/11/4066/s1, Document S1: source code of the tests developed, Spreadsheet S2: complete experimental results.
