# Supplementary material for: Evaluation of Strategies for the Development of Efficient Code for Raspberry Pi Devices
Source: Sensors (Basel). 2018 Nov 21;18(11):4066. doi: 10.3390/s18114066 (PMC6263706; doi:10.3390/s18114066)
Supplement: Supplementary file 1 [file sensors-18-04066-s001.zip › Source code.pdf]

Supplementary Materials: Source Code of "Evaluation of Strategies for the Development of Efficient Code for Raspberry Pi Devices"

Javier Corral-García, José-Luis González-Sánchez and Miguel-Ángel Pérez-Toledano

Contents

Test template used in all experiments 3

T1. Bit fields 4

T2. Boolean return 4

T3. Cascaded function calls 5

T4. Row-major accessing 5

T5. Constructor initialization lists 6

T6. Common subexpression elimination 6

T7. Mapping structures 7

T8. Dead code elimination 8

T9. Exception handling 8

T10. Global variables within loops 8

T11. Function inlining 9

T12. Global variables 9

T13. Constants inside loops 10

T14. Initialization versus assignment 10

T15. Division by a power-of-two denominator 11

T16. Multiplication by a power-of-two factor 11

T17. Integer versus character 11

T18. Loop count down 12

T19. Loop unrolling 12

T20. Passing structures by reference instead of value 13

T21. Pointer aliasing 13

T22. Chains of pointers **14**

T23. Pre-increment versus post-increment **14**

T24. Linear search **15**

T25. Invariant IF statements within loops **15**

Test template used in all experiments

```
#include <cmath>
#include <iostream>
#include "watchtime.h"

#ifdef level
   #define OPTIMIZE __attribute__((optimize(level)))
#else
   #define OPTIMIZE
#endif

const int nms  = 10;         // number of measurements
const int reps = 100000000; // number of times a test is repeated (for a single measurement)

void stats(unsigned times[]){
   unsigned totaltime,meantime,sumsq,sd;
   float testtime;
   totaltime = meantime = sumsq = 0;
   printf("\t\t------------------------------------------------\n");
   for (int i=0; i<nms; i++){
      printf("\t\tTime %d\t%lu ms\n",i+1,times[i]);
      totaltime += times[i];
   }
   meantime = totaltime / nms;
   for (int i=0; i<nms; i++){
      sumsq += (times[i] - meantime) * (times[i] - meantime);
   }
   sd = sqrt(sumsq / (nms-1));
   testtime = ((float)meantime / (float)reps);
   testtime *= 1000000; // ms to ns
   printf("\t\t------------------------------------------------\n");
   printf("\t\tAverage measurement time: %lu ms\n",meantime);
   printf("\t\tStandard deviation: %lu\n",sd);
   printf("\t\t------------------------------------------------\n");
   printf("\t\tAverage test time: %.2f ns\n\n",testtime);
}


int main() {
   int i,j;
   unsigned times[nms],aux;
   Watchtime time;

   printf("\n\nTechnique 1: Bit fields\n\n");
   printf("\tOptimization level %s\n\n",level);

   printf("\tTest 1 (standard code)...\n\n");
   for (i=0; i<nms; i++){
      time.startTime();
      for (j=0; j<reps; j++){
         test1();
      }
      aux = time.getTime();
      times[i] = aux;
   }
   stats(times);

   printf("\tTest 2 (efficient code)...\n\n");
   for (i=0; i<nms; i++){
      time.startTime();
      for (j=0; j<reps; j++){
         test2();
      }
      aux = time.getTime();
      times[i] = aux;
   }
   stats(times);
}
```

T1. Bit fields

```
typedef struct {
  unsigned int bitA : 1;
  unsigned int bitB : 1;
  unsigned int bitC : 1;
  unsigned int bitD : 1;
} BitField;

typedef struct {
  unsigned int bits;
} IntegerBitField;

unsigned int OPTIMIZE getBitField(const BitField *d) {
  return (d->bitA << 0) |
         (d->bitB << 1) |
         (d->bitC << 2) |
         (d->bitD << 3);
}

unsigned int OPTIMIZE getIntegerBitField(const IntegerBitField *d) {
  return d->bits;
}

unsigned int test1() {
     BitField *p;
     return getBitField(p);
}

unsigned int test2() {
     IntegerBitField *p;
     return getIntegerBitField(p);
}
```

T2. Boolean return

```
bool OPTIMIZE getOR(bool argA,bool argB,bool argC,bool argD) {
  return (argA || argB || argC || argD);
}

typedef unsigned int Flags;

#define flagA (1u << 0)
#define flagB (1u << 1)
#define flagC (1u << 2)
#define flagD (1u << 3)

bool OPTIMIZE getFlagsOR(Flags flags) {
  return (flags & (flagA | flagB | flagC | flagD)) != 0;
}

void test1() {
    getOR(1,1,0,0);
}

void test2() {
    getFlagsOR(1100);
}
```

T3. Cascaded function calls

```
int const N = 20;
int a[N];

class Class {
   private:
      int data;
   public:
      void setData(int data);
      int  getData();
};

void Class::setData(int data) {
    Class::data = data;
}

int Class::getData(){
    return data;
}

void OPTIMIZE test1(Class *c){
    for (int i=0; i<N; i++){
        if (c->getData()==1){
           a[i] = 0;
        }
    }
}

void OPTIMIZE test2(Class *c){
    int data = c->getData();
    for (int i=0; i<N; i++){
        if (data==1){
           a[i] = 0;
        }
    }
}
```

T4. Row-major accessing

```
int const N = 60;
int array[N][N];

void OPTIMIZE test1(){
  for (int j=0; j<N; j++)
      for (int i=0; i<N; i++)
          array[i][j] = 0;
}

void OPTIMIZE test2(){
  for (int i=0; i<N; i++)
      for (int j=0; j<N; j++)
          array[i][j] = 0;
}
```

T5. Constructor initialization lists

```
using namespace std;

class ClassA {
   private:
      string dataA;
      string dataB;
      int    dataC;
      int    dataD;
   public:
      ClassA(string data1, string data2, int data3, int data4);
};

OPTIMIZE ClassA::ClassA(string data1, string data2, int data3, int data4) {
    dataA = data1;
    dataB = data2;
    dataC = data3;
    dataD = data4;
}

class ClassB {
   private:
      string dataA;
      string dataB;
      int    dataC;
      int    dataD;
   public:
      ClassB(string data1, string data2, int data3, int data4);
};

OPTIMIZE ClassB::ClassB(string data1, string data2, int data3, int data4):
        dataA(data1), dataB(data2), dataC(data3), dataD(data4) {
}

void OPTIMIZE test1(){
    ClassA c("data1","data2",1,1);
}

void OPTIMIZE test2(){
    ClassB c("data1","data2",1,1);
}
```

T6. Common subexpression elimination

```
int x=100;
int i,j;

void OPTIMIZE test1(){
    i = x + sqrt(16384) + 1;
    j = x + sqrt(16384);
}

void OPTIMIZE test2(){
    int aux = x + sqrt(16384);
    i = aux + 1;
    j = aux;
}
```

T7. Mapping structures

```c
#define NELEMS(a) ((int) (sizeof(a) / sizeof(a[0])))

static const struct {
    const char data[7]; /* NB. PIC */
    int        value;
} map[] = {
    { "AAAAAA", 1 },
    { "BBBBBB", 2 },
    { "CCCCCC", 3 },
    { "DDDDDD", 4 },
    { "EEEEEE", 5 },
    { "FFFFFF", 6 },
    { "GGGGGG", 7 },
    { "HHHHHH", 8 },
    { "IIIIII", 9 },
    { "JJJJJJ", 9 },
    { "KKKKKK", 9 },
    { "LLLLLL", 9 },
    { "MMMMMM", 9 },
    { "OOOOOO", 9 },
    { "PPPPPP", 9 },
    { "QQQQQQ", 9 },
    { "RRRRRR", 9 },
    { "SSSSSS", 9 },
    { "TTTTTT", 9 }
};

int OPTIMIZE dataToValue(const char *data) {
    if      (strcmp(data, "AAAAAA") == 0) return 1;
    else if (strcmp(data, "BBBBBB") == 0) return 2;
    else if (strcmp(data, "CCCCCC") == 0) return 3;
    else if (strcmp(data, "DDDDDD") == 0) return 4;
    else if (strcmp(data, "EEEEEE") == 0) return 5;
    else if (strcmp(data, "FFFFFF") == 0) return 6;
    else if (strcmp(data, "GGGGGG") == 0) return 7;
    else if (strcmp(data, "HHHHHH") == 0) return 8;
    else if (strcmp(data, "IIIIII") == 0) return 9;
    else if (strcmp(data, "JJJJJJ") == 0) return 10;
    else if (strcmp(data, "KKKKKK") == 0) return 11;
    else if (strcmp(data, "LLLLLL") == 0) return 12;
    else if (strcmp(data, "MMMMMM") == 0) return 13;
    else if (strcmp(data, "NNNNNN") == 0) return 14;
    else if (strcmp(data, "OOOOOO") == 0) return 15;
    else if (strcmp(data, "PPPPPP") == 0) return 16;
    else if (strcmp(data, "QQQQQQ") == 0) return 17;
    else if (strcmp(data, "RRRRRR") == 0) return 18;
    else if (strcmp(data, "SSSSSS") == 0) return 19;
    else if (strcmp(data, "TTTTTT") == 0) return 20;
    else return -1; /* default case */
}

int OPTIMIZE dataToValue2(const char *data) {
    for (int i = 0; i < NELEMS(map); i++)
        if (strcmp(data, map[i].data) == 0)
            return map[i].value;
    return -1; // default case
}

int test1() {
    return dataToValue("TTTTTT");
}

int test2() {
    return dataToValue2("TTTTTT");
}
```

T8. Dead code elimination

```
int global;

void OPTIMIZE test1(){
  int i;
  i = 1;          // dead store
  global = 1;     // dead store
  global = 2;
  return;
  global = 3;     // unreachable
}

void OPTIMIZE test2(){
  global = 2;
  return;
}
```

T9. Exception handling

```
using namespace std;

class myexception: public exception {
} myex;

int OPTIMIZE test1(){
   int num = 100;
   for (int i=0; i<1; i++){
      try{
           if (num == 100) {
                throw myex;
           }
      } catch (exception& e){
      }
   }
   return 0;
}

int OPTIMIZE test2(){
   int num = 100;
   for (int i=0; i<1; i++){
          if (num != 100) {
                continue;
           }
   }
   return 0;
}
```

T10. Global variables within loops

```
int const N = 20;
int a[N];
int sum;

void initializeArray(int N){
  for (int i=0; i<N; i++){
     a[i] = i;
  }
}

void OPTIMIZE test1(){
  sum = 0;
  for (int i=0; i<N; i++)
    sum += a[i];
}

void OPTIMIZE test2(){
  int t = 0;
  for (int i=0; i<N; i++)
     t += a[i];
  sum = t;
}
```

T11. Function inlining

```
int OPTIMIZE add (int x, int y) {
   return x + y;
}

int OPTIMIZE sub(int x, int y) {
  return add (x, -y);
}

int OPTIMIZE sub2(int x, int y) {
  return x + -y;
}

void test1(){
   sub(10,5);
}

void test2(){
   sub2(10,5);
}
```

T12. Global variables

```
int value;

int f() {
   return 512;
}

void OPTIMIZE test1() {
  for (int i=0; i<50; i++) {
     value += f();
  }
}

void OPTIMIZE test2() {
  int aux = value;
  for (int i=0; i<50; i++) {
     aux += f();
  }
  value = aux;
}
```

T13. Constants inside loops

```
#define IDENTIFIER_A 2
#define IDENTIFIER_B 1
#define IDENTIFIER_C 3

typedef struct {
  unsigned int value;
} Structure;

void aux1(int i){
   i++;
}
void aux2(int i){
   i++;
}
void aux3(int i){
   i++;
}


void OPTIMIZE test1(int N) {
  int i;
  Structure t,*pt;
  pt = &t;

  pt->value &= IDENTIFIER_C;

  for (i=0; i<N; i++) {
    if (pt->value & IDENTIFIER_A)
      aux1(i);
    else if (pt->value & IDENTIFIER_B)
      aux2(i);
    else
      aux3(i);
   }
}

void OPTIMIZE test2(int N) {
  int i;
  Structure t,*pt;
  pt = &t;

  pt->value &= IDENTIFIER_C;

  if (pt->value & IDENTIFIER_A) {
    for (i=0; i<N; i++)
        aux1(i);
  }else if (pt->value & IDENTIFIER_B) {
    for (i=0; i<N; i++)
        aux2(i);
  }else {
    for (i=0; i<N; i++)
        aux3(i);
  }
}
```

T14. Initialization versus assignment

```
void OPTIMIZE test1() {
    std::complex<double> mycomplex;
    mycomplex = (3.14);
}

void OPTIMIZE test2() {
    std::complex<double> mycomplex(3.14);
}
```

T15. Division by a power-of-two denominator

```
int OPTIMIZE divide (unsigned int i) {
  return i / 1024;
}

int OPTIMIZE divide2 (unsigned int i) {
  return i >> 10;
}

void test1(){
   divide(100000000);
}

void test2(){
   divide2(100000000);
}
```

T16. Multiplication by a power-of-two factor

```
int OPTIMIZE multiply (int i) {
  return i * 1024;
}

int OPTIMIZE multiply2 (unsigned int i) {
  return i >> 10;
}

void test1(){
   multiply(100000000);
}

void test2(){
   multiply2(100000000);
}
```

T17. Integer versus character

```
char OPTIMIZE sum_char(char a, char b, char c, char d, char e) {
    return a+b+c+d+e;
}

int OPTIMIZE sum_int(int a, int b, int c, int d, int e) {
    return a+b+c+d+e;
}

void test1(){
    sum_char(1,2,3,4,5);
}

void test2(){
    sum_int(1,2,3,4,5);
}
```

T18. Loop count down

```
int const N = 100;
int a[N];

void OPTIMIZE test1(){
  for (int i=0; i<N; i++) {
      a[i]=i;
  }
}

void OPTIMIZE test2(){
   int i = N+1;
   while (--i) {
      a[i] = i;
   }
}
```

T19. Loop unrolling

```
const int N = 50;
int array[N];

void OPTIMIZE initialization1(){
   int i;
   for (i=0; i<N; i++){
     array[i] = 0;
   }
}

void OPTIMIZE initialization2(){
   int i;
   for (i=0; i<N; i+=5){
     array[i]   = 0;
     array[i+1] = 0;
     array[i+2] = 0;
     array[i+3] = 0;
     array[i+4] = 0;
   }
}

void test1(){
    initialization1();
}

void test2(){
    initialization2();
}
```

T20. Passing structures by reference instead of value

```
using namespace std;

typedef struct {
    int array[10];
    int value;
}  Structure;

class Class {
   private:
      string    data_a ;
      string    data_b;
      Structure structure;
   public:
      Class(string data1, string data2, int i);
      int getIndex();
};

OPTIMIZE Class::Class(string data1, string data2, int i) {
    data_a = data1;
    data_b = data2;
    structure.value = i;
}

int OPTIMIZE Class::getIndex() {
    return structure.value;
}

int OPTIMIZE test1(Class value){
    return value.getIndex();
}

int OPTIMIZE test2(Class *reference){
    return reference->getIndex();
}
```

T21. Pointer aliasing

```
int a,b,c,d,e;
int *pa = &a;
int *pb = &b;
int *pc = &c;
int *pd = &d;
int *pe = &e;

void OPTIMIZE pointersA(int *t1, int *t2, int *t3, int *t4, int *step) {
    *t1 += *step;
    *t2 += *step;
    *t3 += *step;
    *t4 += *step;
}

void OPTIMIZE pointersB(int *t1, int *t2, int *t3, int *t4, int *step) {
    int s = *step;
    *t1 += s;
    *t2 += s;
    *t3 += s;
    *t4 += s;
}

void test1() {
     pointersA(pa,pb,pc,pd,pe);
}

void test2() {
     pointersB(pa,pb,pc,pd,pe);
}
```

T22. Chains of pointers

```
typedef struct { int a,b,c,d,e; } Values;
typedef struct { Values *values; } Structure;

Structure structure,*pstructure;
Values values;

void OPTIMIZE test1() {
  structure.values = &values;
  pstructure = &structure;

  pstructure->values->a = 0;
  pstructure->values->b = 0;
  pstructure->values->c = 0;
  pstructure->values->d = 0;
  pstructure->values->e = 0;
}

void OPTIMIZE test2() {
  structure.values = &values;
  pstructure = &structure;

  Values *aux = pstructure->values;
  aux->a = 0;
  aux->b = 0;
  aux->c = 0;
  aux->d = 0;
  aux->e = 0;
}
```

T23. Pre-increment versus post-increment

```
int const N = 200;
int array[N+1];

void OPTIMIZE test1(){
   for (int i=0; i<N;){
        array[i] = i++;
   }
}

void OPTIMIZE test2(){
   for (int i=0; i<N;){
        array[i] = ++i;
   }
}
```

T24. Linear search

```
int const N = 100;
int list [N];
int *plist;

void OPTIMIZE inicialize(int *list, int N){
  for (int i=0; i<N; i++){
     list[i] = i;
  }
}

int OPTIMIZE search1(int *list, int N, int want) {
  int i;
  for (i = 0; i < N; i++)
    if (list[i] == want)
      return i;
  return -1;
}

int OPTIMIZE search2(int *list, int N, int want) {
  int i;
  list[N] = want;
  i = 0;
  while (list[i] != want)
    i++;
  if (i == N)
    return -1;
  return i;
}

int test1(){
   plist = list;
   return search1(plist,N,98);
}

int test2(){
   plist = list;
   return search2(plist,N,98);
}
```

T25. Invariant IF statements within loops

```
int x=0;

int const N = 100;
int a[N];
int b[N];

void OPTIMIZE test1(){
  for (int i=0; i<N; i++)
    if (x==1)
      a[i] = 0;
    else
      b[i] = 0;
}

void OPTIMIZE test2(){
  if (x==1)
     for (int i=0; i<N; i++)
       a[i] = 0;
  else
     for (int i=0; i<N; i++)
       b[i] = 0;
}
```
